# A minimally invasive optical trapping system to understand cellular interactions at onset of an immune response

**DOI:** 10.1371/journal.pone.0188581

**Published:** 2017-12-08

**Authors:** David G. Glass, Niall McAlinden, Owain R. Millington, Amanda J. Wright

**Affiliations:** 1 Institute of Photonics, SUPA, The University of Strathclyde, Glasgow, United Kingdom; 2 Strathclyde Institute of Pharmacy and Biomedical Sciences, The University of Strathclyde, Glasgow, United Kingdom; 3 Optics and Photonics Group, Department of Electrical and Electronic Engineering, The University of Nottingham, Nottingham, United Kingdom; University of Heidelberg Medical School, GERMANY

## Abstract

T-cells and antigen presenting cells are an essential part of the adaptive immune response system and how they interact is crucial in how the body effectively fights infection or responds to vaccines. Much of the experimental work studying interaction forces between cells has looked at the average properties of bulk samples of cells or applied microscopy to image the dynamic contact between these cells. In this paper we present a novel optical trapping technique for interrogating the force of this interaction and measuring relative interaction forces at the single-cell level. A triple-spot optical trap is used to directly manipulate the cells of interest without introducing foreign bodies such as beads to the system. The optical trap is used to directly control the initiation of cell-cell contact and, subsequently to terminate the interaction at a defined time point. The laser beam power required to separate immune cell pairs is determined and correlates with the force applied by the optical trap. As proof of concept, the antigen-specific increase in interaction force between a dendritic cell and a specific T-cell is demonstrated. Furthermore, it is demonstrated that this interaction force is completely abrogated when T-cell signalling is blocked. As a result the potential of using optical trapping to interrogate cellular interactions at the single cell level without the need to introduce foreign bodies such as beads is clearly demonstrated.

## Introduction

Using a high numerical aperture microscope objective lens and a laser beam, optical trapping provides three dimensional control and manipulation of objects ranging in size from hundreds of nanometers to tens of microns [1]. Since the first demonstration of optical trapping and manipulation of viruses and bacteria in the late 1980s, optical trapping has emerged as a powerful tool with many applications in the life sciences. Applications range from manipulation and positional control, to the measurement of forces within the pico-Newton range, a magnitude that is comparable to many biological functions [2, 3]. In particular it has proven to be an incredibly useful non-invasive tool for probing and understanding cells at the single-cell level, as opposed to analyzing bulk samples, providing additional insight into the behavior and function of individual cells [4]. Holographic optical traps can provide re-configurable positional control of several trap positions simultaneously [5], allowing cell orientation and cell contact time to be controlled and giving precise control over multiple particles. Using an optical trap it is possible to control the length of a specific interaction and ensure that the interaction studied is the initial contact between a cell pair. Optical trapping provides an excellent route to not only control but also to quantify relative interaction forces on the pico-Newton scale, making them ideal for initial stage cell pair interaction studies [4].

Competing technologies capable of studying the relative interaction force between single cell pairs include atomic force microscopy (AFM), magnetic tweezers and micropipette aspiration [6–8]. For cell-cell interaction measurements using an AFM a cell is attached to a cantilever tip and the deflection of the tip monitored as the cell is brought into contact with a neighboring cell. Magnetic tweezers inject exogenous ferromagnetic beads into a sample and observe the motion of the beads in response to directional magnetic fields. The beads themselves have to be re-magnetized after a period of time making them unsuitable for long term measurements. When using micropipette aspiration a cell is attached to the end of a micropipette using suction and the deformation and response of this cell monitored in relation to neighboring cells [8]. In terms of measurement range optical tweezers are unique covering a lower range of forces then competing techniques, operating between 0.1–100 pN compared to ~5–10,000 pN for AFM and 2–50 pN for magnetic tweezers [6, 7]. Perhaps most importantly, optical tweezers do not require mechanical contact with the cell of interest, for example via a cantilever or micro-pipette, and therefore greatly reduce the possibility of cell and sample damage during measurement. This has an added advantage that, for the periods of time when the optical trapping laser is turned off, the cell is free to interact without physical attachment and can therefore scan target cells freely during the interaction period, more closely replicating the *in vivo* situation. Wei *et al*. and Miller *et*. *al*. have demonstrated the importance of T cells to be able to scan the surface of neighboring cells during the interaction processes [9, 10].

T-cells and antigen presenting cells (APC) are an important group of cells that form part of our adaptive immune system, responsible for clearing an infection and establishing immunological memory. How these cells interact with each other, and the force or duration of their interaction, is known to determine the type of immune response and whether the body successfully fights a particular pathogen or disease [11, 12]. *In vivo* imaging of these interactions has revealed the dynamic nature of this process [13] and the difference the dose of antigen or the duration of the interaction can have on the development of an effective immune response [14, 15]. To date there are conflicting studies on what effect the duration and strength of the cellular interaction has on the efficiency of T-cell activation and the development of an immune synapse [16, 17]. A well calibrated optical trapping system therefore provides the ideal route to study and interrogate the early stages of these interactions at the single-cell level.

In 1991 Seeger *et al*. showed that optical trapping could be a useful tool in immunology to observe the first stages of cellular interactions, improving on previous techniques requiring time-consuming sample preparation which meant that the initial stages of interaction were often missed [18]. Seeger *et al*. were interested in the interaction between natural killer cells and cancer cells and used an optical trap to move a single natural killer cell to its target [18]. Subsequently, optical trapping has been used in conjunction with optical sectioning microscopy to orientate an immune cell pair and place the immunological synapse in the image plane of a confocal microscope [19]. Studies have used optical trapping to probe the sensitivity of a T-cell to stimulation using an antibody coupled to exogenous beads to examine the cell signaling associated with cell activation [18–24]. The response of the T-cell to non-specific activation was monitored by imaging the calcium flux [17, 20] or observing the cells morphological response [21]. Morrison *et al*. have used optical trapping to investigate the role an adhesion receptor called Beta2-integrin has on regulating an immune response by measuring the interaction force between T-cells and dendritic cells [25].

The work presented here builds on these initial studies and represents a significant advance over previous systems employed to manipulate T-cells [26]. We combine the capacity to manipulate interactions in real-time whilst also determine the relative interaction forces associated with antigen recognition. We present a reliable, versatile methodology for interrogating the interaction force between T-cells and dendritic cells and observe changes in the relative force due to pharmacological inhibition of cell signaling. Importantly, not only does our approach study the relative interaction force at a single cell level but we directly trap the T-cells themselves removing the need to introduce exogenous beads and foreign bodies to the sample which run the risk of artificially perturbing the system we are trying to measure.

## Methodology

**Fig 1:**
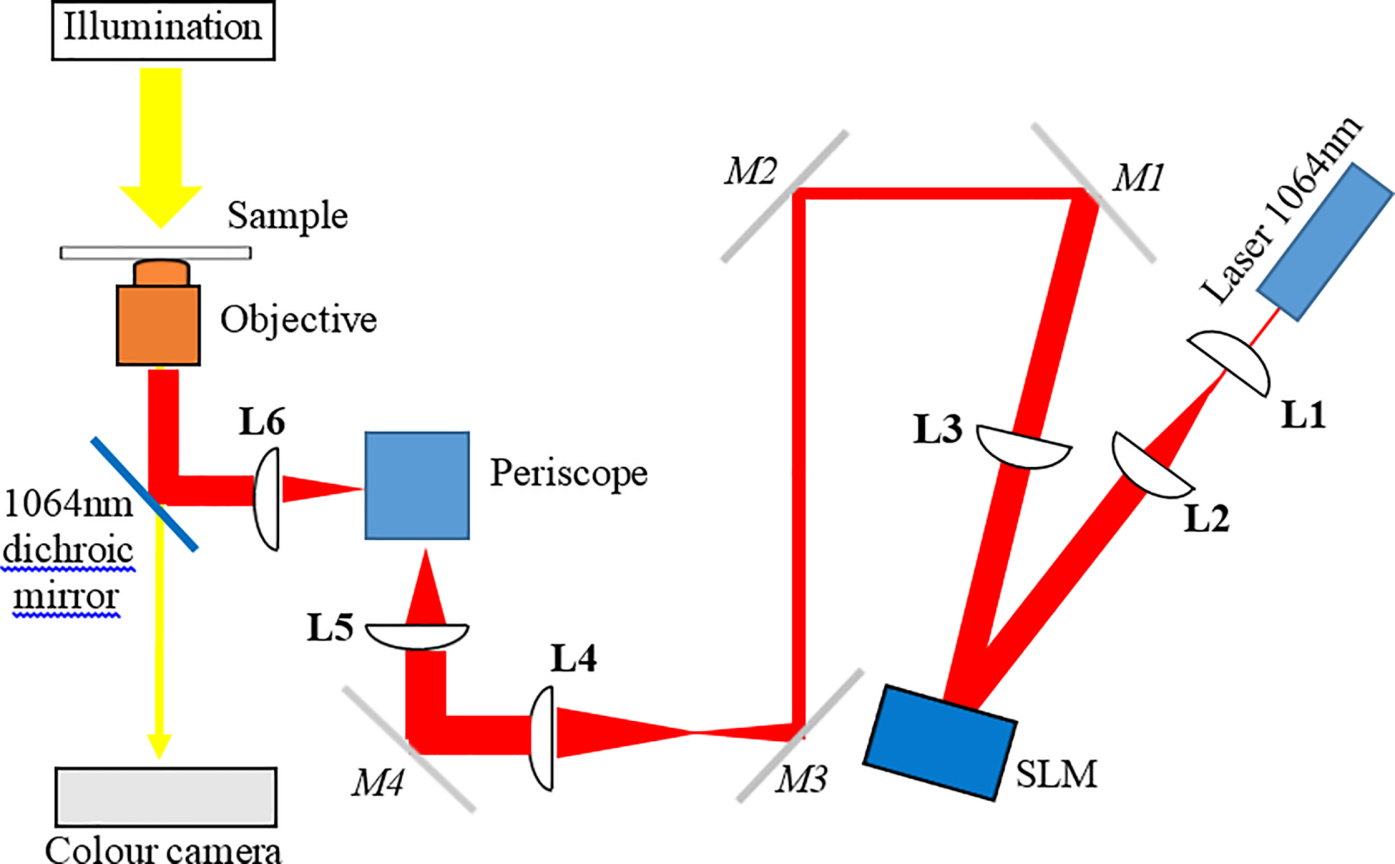
A schematic of the optical trapping system. A 1064 nm wavelength laser beam was expanded using lenses L1 and L2 to fill the spatial light modulator (SLM) display. The SLM is re-imaged onto mirror 5 (M5), using lenses L3 and L4, and then from M4 onto the back aperture of the microscope objective lens, using lenses L5 and L6. A periscope directs the light into the main body of the microscope and a dichroic mirror reflects the 1064nm light to the sample, transmitting the white light used for imaging. M1-4 are beam steering mirrors.

The optical trapping system consisted of a Ventus IR, 3 W, TEM00, continuous wave laser (Laser Quantum, UK) with a wavelength of 1064 nm that was expanded to just overfill a spatial light modulator (SLM; Boulder Nonlinear Systems, USA). The SLM consisted of 512x512 individually addressable pixels that altered the phase of the light and projected a hologram onto the back aperture of a microscope objective using a technique known as holographic optical tweezers or HOT [5, 27]. For the experiments conducted here the SLM was used to project a diffraction pattern onto the back aperture of the microscope objective, altering the spacing and angle of the diffraction pattern resulted in changing the position of the optical trap in x and y in the sample plane. The system was designed round a Nikon TE2000-U inverted microscope (Nikon, UK) equipped with a heated stage (Brunel Microscopes, UK) to maintain temperature at 37°C throughout the experiment. The microscope had a high-precision, computer controlled, sample stage (Advanced Scientific Instruments, USA). The microscope objective was a ×100 oil immersion objective with a high numerical aperture (NA = 1.3) required for optical trapping. The laser power was manually adjusted and measured at the back aperture of the objective. To calculate the power at the sample a 39% loss was assumed through the objective lens [28]. Images were acquired either with a QCam colour camera (Qimaging, Canada) or a monochrome Genie camera (TeledyneDALSA, Canada). A schematic of the optical system can be seen in Fig 1.

**Fig 2:**
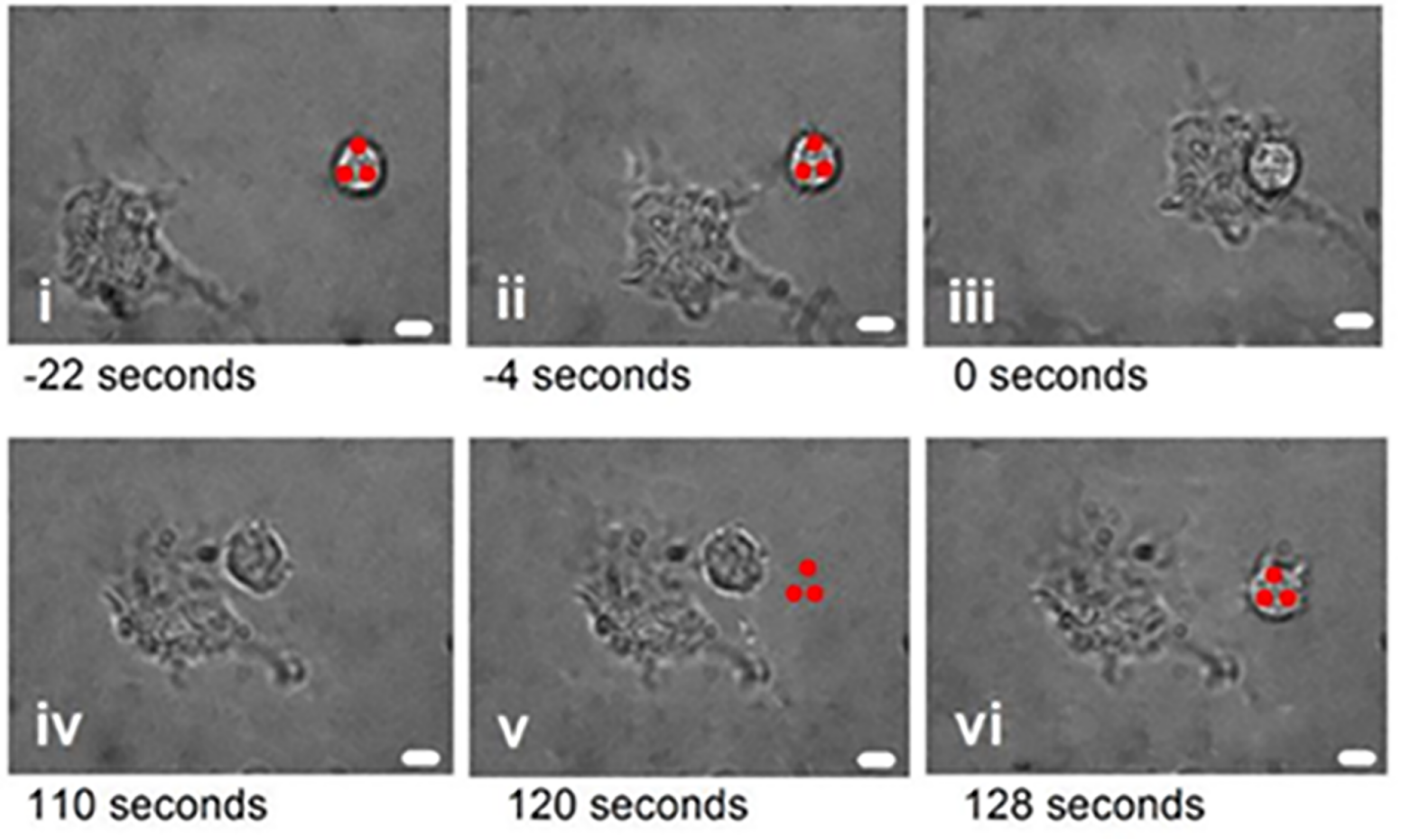
The methodology used to study interaction forces between T-cells and dendritic cells. The optical trap (red spots) was used to bring the cells into contact (i). As soon as contact was made (ii), the trap was blocked and the cells left to interact for a given period of time (iii—iv). Subsequently, the trap was re-instated 5 μm from the T cell (v) and the laser beam power incrementally increased until the T-cell was released from the dendritic cell and held by the optical trap, breaking cell contact (vi). The scale bar represents 4μm.

The primary T-cells used were measured to be 6 μm in diameter (± 0.16 μm; n = 36 cells) and generally spherical when in suspension, making them ideal candidates for optical trapping. In the technique presented here, the T-cell itself is optically trapped and no exogenous beads are added to the system. When trapped with a single Gaussian beam, focal spot ~ 500 nm in diameter, the T-cells are liable to roll within the trapping volume and their orientation can change throughout an experiment or individual measurement. This is due to a high refractive index feature of the cell being trapped rather than the cell as a whole. To fix the position of the trapped T-cell and prevent any re-orientation and rolling during an experiment we used a triple-spot trapping beam, similar to that described in reference [29]. To achieve this the laser beam was split into three separate beams using the SLM and the three focal points, each nominally with Gaussian intensity distributions, were positioned just inside the cell membrane separated by approximately 5 μm (see Fig 2).

The viability of optically trapped T-cells have been previously confirmed by McAlinden *et al*. using propidium iodide as a fluorescence maker for viability [30]. Here it was shown that T-cells can be optical trapped with a triple-spot trap for over an hour using a 1064 nm wavelength laser beam and ~30 mW of power at the sample without showing any sign of damage.

Dendritic cells were generated from bone marrow of C57BL/6 mice by culture in RPMI supplemented with L-Glutamine (2 mM), penicillin (100 μg/ml), streptomycin (100 μg/ml), 10% fetal calf serum (all Invitrogen, UK) and 10% of culture supernatant from X63 myeloma cells transfected with mouse GM-CSF, as previously described [31]. Mature dendritic cells were plated at a final concentration of 1x10^4^/ml and antigen pulsed with 1 mg/ml ovalbumin (OVA; Sigma-Aldrich, UK) and/or stimulated with 1 μg/ml LPS (Sigma-Aldrich) overnight at 37°C in 5% CO2. Sample slides were used (ibidi, Germany) with pairs of chambers connected via a flow channel to ensure the T-cell was interacting with the APC for the first time and had not been in previous contact. Dendritic cells were added into one well and cultured overnight, allowing them to adhere to the bottom coverslip of the imaging chamber. CD4^+^ OVA-specific T-cells were isolated from OT-II mice using negative selection (Miltenyi Biotec, UK) and re-suspended at 1x10^4^/ml for addition to the imaging chamber. In the later experiments the T-cells were treated for 1 hour with 10 μM FR180204 (ERK inhibitor [32]; Tocris Bioscience, UK). The OT-II transgenic mice were originally from Charles River Laboratories (USA), and were maintained as colony at the Biological Procedure Unit (BPU) of Strathclyde Institute of Pharmacy and Biomedical Sciences (SIPBS). They express an alpha and beta chain TCR that pairs with CD4 co-receptor and is specific for the Ovalbumin (OVA) 323–339 peptide in the context of I-Ab. All lymphocyte preparations were prepared from these mice.

Fig 2 shows a series of images outlining the experimental procedure used to characterize the interaction force between immune cell pairs. First, the optical trap selects and isolates the T-cell of interest and the sample stage is moved to bring the T-cell into contact with a dendritic cell, initiating cell contact. After a pre-determined amount of time the optical trap is re-instated 5μm from the interacting T-cell and the laser beam power gradually increased. Increasing the laser power increases the external force acting on the T-cell and eventually the cellular contact is broken and T-cell again held in the optical trap. The power required to break the cellular contact is attributed to a maximum optical trapping force and this provides a relative cellular interaction force at the point when the T-cell is released from the dendritic cell. It is important that the optical trapping force is pre-calibrated and the relationship between laser beam power and total trapping force accurately known. The method used to calibrate the trap will be discussed in detail in the next section.

## Results

### Calibration of the triple spot optical trap

**Fig 3:**
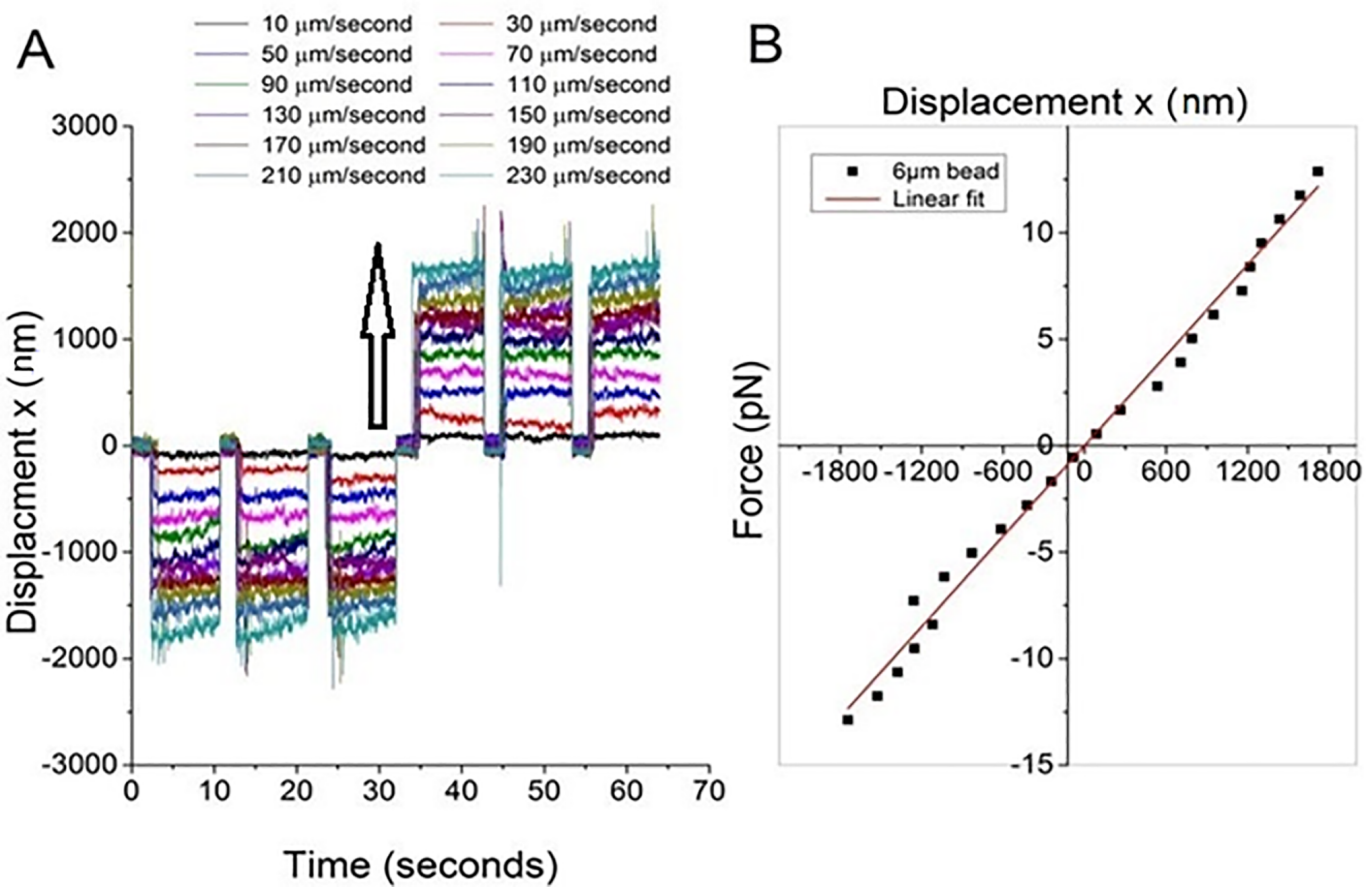
Characterizing the relationship between displacement from equilibrium and force for a 6μm bead trapped using a triple spot optical trap. a) shows the relationship between displacement versus time for a trapped bead when exposed to an increasing external viscous drag force created by moving the sample stage with increasing speed (the arrow represents speed increasing from 10 μms^-1^ to 230 μms^-1^). b) shows the average bead displacement, taken from plot 3a, against applied external force in pN showing a linear relationship between force and displacement when the displacement < 2000 nm. Data taken at a fixed laser beam power.

We have previously demonstrated the ability of a triple-spot trap, with three trapping sites placed just inside the cell wall, to reduce cell roll and re-orientation during measurement [29]. For the experiments presented here it is important that the relationship between trapping force and laser beam power is correctly calibrated so that the cellular interaction force can be inferred from the laser beam power required to separate a cell pair. Several well-established approaches exist that allow the trap strength/trapping force to be measured and the choice of approach will depend upon the distances or displacements from the trap center. For small displacements of an object from trap centre it is possible to model the trap as a weight on a spring and apply Hooke’s Law where *F* = −*kx* with *F* the optical trapping force, *k* the trap strength or spring constant and *x* the displacement of the object from trap center. To determine the distance over which Hooke’s Law applies when using the triple spot trap, a 6μm diameter polystyrene bead was optically trapped (a size comparable to the T-cells of interest) and an external viscous drag force applied by moving the sample stage at a known and constant speed. A center of mass tracking algorithm was used to track the position of the bead in response to the external force, see Fig 3A. For the data presented in Fig 3, the total laser beam power was 25mW at the sample, and this was split between the three trapping sites. The sample stage speeds applied were selected to be below the speed at which the bead would be released from the trap.

Fig 3A shows twelve traces of trap position versus time relating to sample stage speeds increasing from 10 μms^-1^ to 230 μms^-1^ when the stage is first moved in the negative x direction for 3 cycles and then in a positive x direction for 3 cycles. Fig 3B presents the external viscous drag force, determined using Stokes’ Law, versus the average displacement of the trapped bead from equilibrium position. The sample stage was moved in a negative x direction and then positive x direction to increase the number of points included in Fig 3B. The trap depth (i.e. the distance between the trapped bead and the bottom coverslip) was kept constant throughout. Beyond 2μm displacement from equilibrium (equivalent to 1/3 of the beads diameter) and a sample stage speed 230 μms^-1^ the bead no longer remains trapped, the relationship between force and displacement is no longer linear and Hooke’s law can no longer be applied. A similar approach to determining the linear trapping region was taken by Simmons et al [33]. In our experiment, the trap was placed 5μm from the interacting immune cells and we are therefore working in a regime where Hooke’s Law does not apply and methods such as the equipartition method [1] cannot be applied. When applied to optical trapping, the equipartition method equates the thermal energy in the system to the potential energy stored in the optical trap using Hooke’s Law and therefore assumes that displacement from trap center is linearly proportional to force. Instead the viscous drag force method [33, 34] was used to measure the maximum optical trapping force.

**Fig 4:**
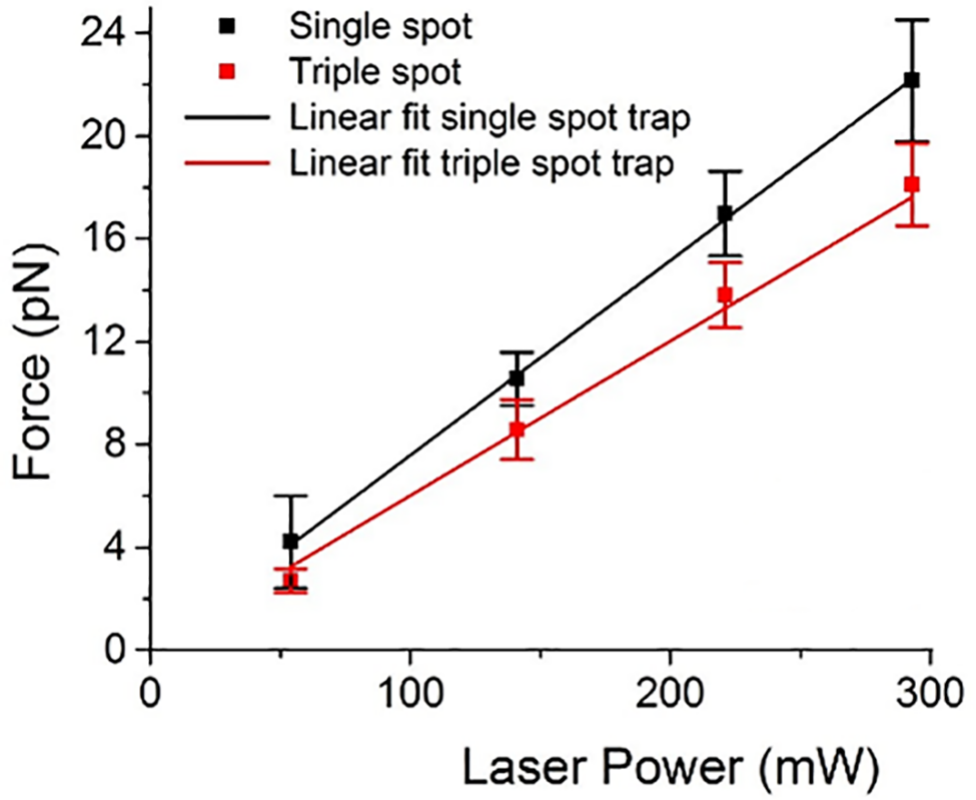
Laser beam power at the sample versus optical trapping force for a T-cell trapped using a single-spot (black squares) and triple-spot (red squares) trap. Trap depth was kept constant at 10 μm above the coverslip surface, and each point represents the mean ± standard deviation of 36 individual measurements.

**Fig 5:**
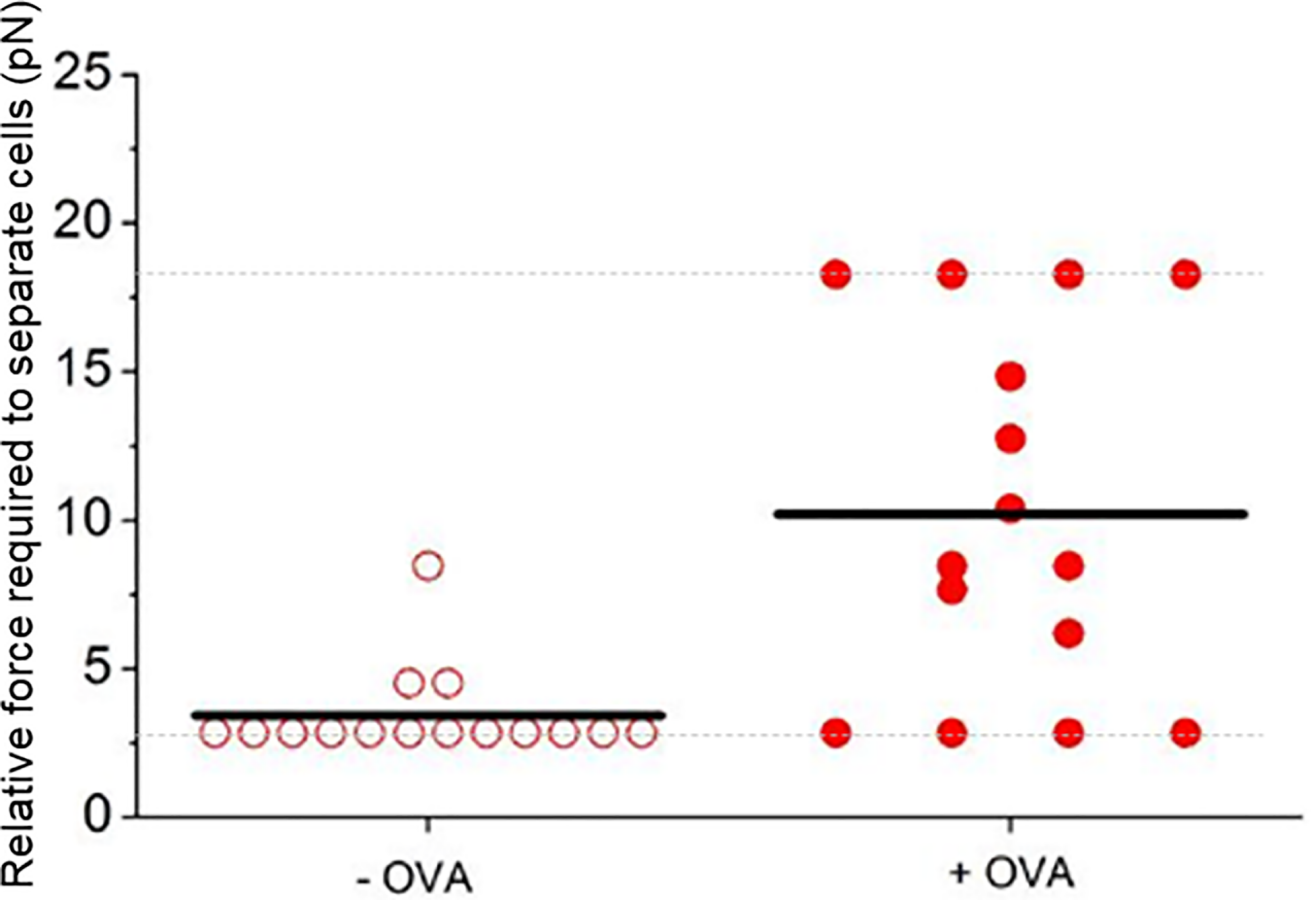
Antigen-specific increase in interaction force between dendritic cells and T-cells. Bone marrow-derived dendritic cells were pulsed with 1 mg/ml OVA (filled symbols) before addition of OVA-specific OT-II T-cells. Control cells remained un-pulsed (empty symbols). The relative force required to separate T-cells and dendritic cells after 30 seconds of interaction was determined for 15 cell pairs using the approach outlined in Fig 2. The black lines represent the median average of the measurements. The dotted grey line the minimum and maximum measurement range. A significant difference in relative interaction force is seen as evidenced by the Mann Whitney test (p ≤ 0.005).

**Fig 6:**
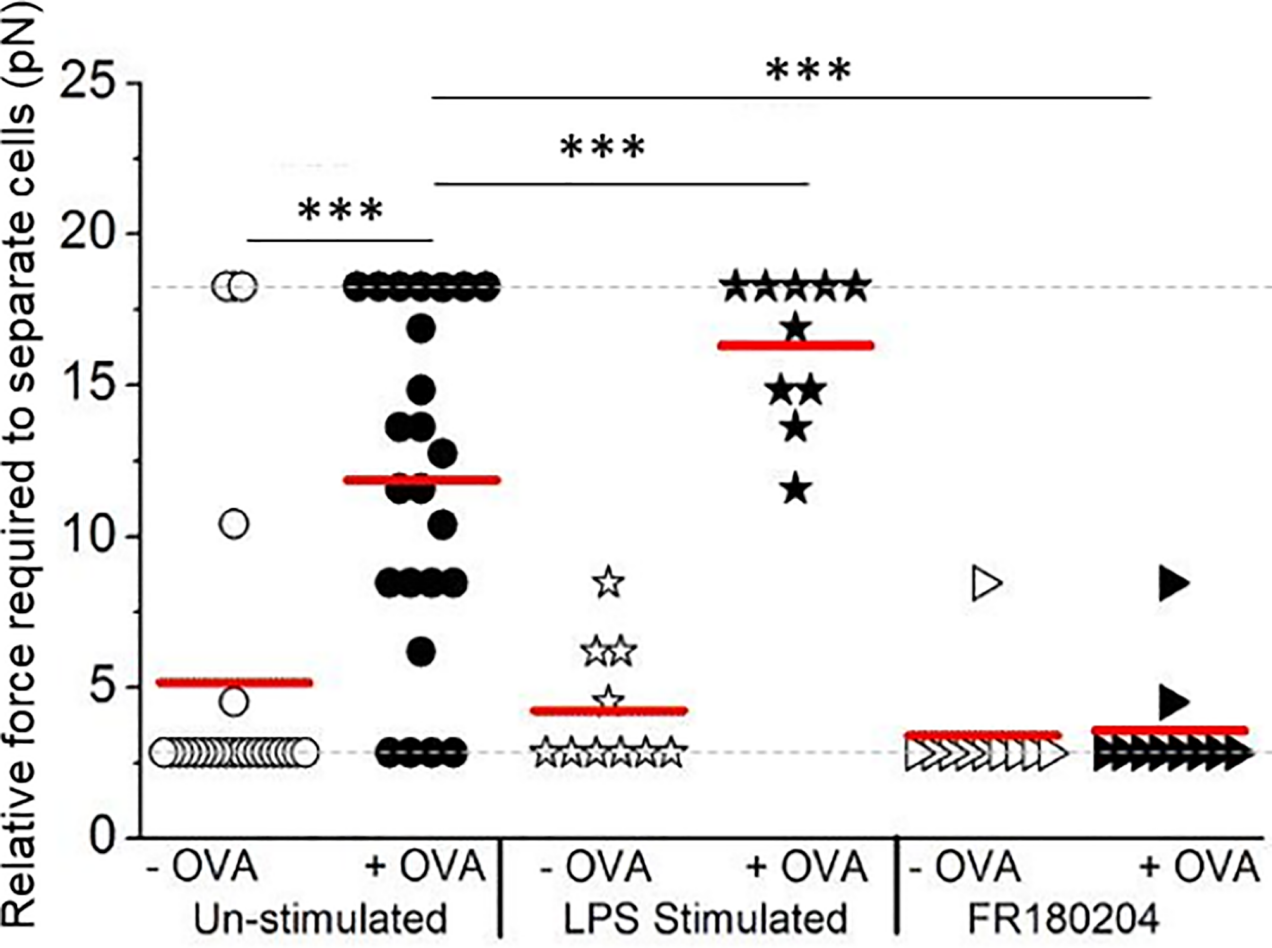
Interrogating changes in the cellular interaction force as a result of therapeutic intervention. Cell interaction forces measured as in Fig 5 except that the dendritic cells were pre-stimulated with LPS or the T-cells were pre-treated with 10 μM of ERK-inhibitor FR180204 prior to analysis. After the optical trap was used to establish contact, cells were allowed to remain in contact for 120 seconds before measuring the relative interaction force. The plot shows the relative force required to separate the cell pairs with the median indicated with a red line, the dotted grey line represents the detection range. (*** *p* ≤ 0.0005, by Mann Whitney test).

For the viscous drag force method, at a fixed laser beam power, the sample stage velocity is increased until the cell or bead is released from the optical trap and at which point the trapping force is equated to the viscous drag force determined using Stokes’ Law where *F* = −6*πηaV* and *η* the viscosity of the surrounding medium, *a* the diameter of the trapped object and *V* the relative speed between the sample stage and trapped object. The sample stage was moved as opposed to the trapped cell since the optical trapping force is known to vary across the field of view, particularly when using an SLM due to optical aberrations and varying diffraction efficiency. Fig 4 compares the relationship between laser beam power at the sample and trapping force for a single-spot trap and a triple-spot trap when trapping a T-cell, each data point representing an average of 36 readings. The trap depth was kept fixed at 5 μm above the coverslip to ensure that the trapping force did not vary due to changing proximity to a boundary. For both the single-spot and the triple-spot trap the trapping force increased linearly with laser beam power as would be expected. The range of laser beam powers available places an upper and lower limit on the force measurement. Since some interactions exceeded the maximum force we could apply and the contact could not be broken we present the median instead of the mean for each data set shown in Figs 5 and 6.

### Interrogating the interaction force between individual T-cells and dendritic cells

To confirm the ability of the technique to manipulate cell-cell interactions and infer a relative interaction force, we used dendritic cells to present antigen to T-cells with known antigen-specificity. OVA-specific T-cells are commonly used in immunological studies and are routinely used in imaging experiments. Fig 5 compares the relative interaction force between individual T-cells and dendritic cells after 30 seconds of contact time for 15 cell pairs for each case.

In the absence of antigen (-OVA group), the cellular interactions were relatively easy to disrupt and a median trapping force of 3.3 pN (± 1.4 pN) was required to separate the T-cell from the dendritic cell. Conversely, when T-cells were presented with their cognate antigen, a significantly greater force was required to separate cell pairs (median force of 8.5 pN ± 5.7 pN). This represents a 2.6 fold increase in relative cellular interaction force for the with antigen case, when the cognate antigen was present on the dendritic cells, compared to the without antigen case.

Finally, the potential of this single cell-pair approach for investigating the mechanism of action of therapeutic candidates was assessed. For the first intervention the dendritic cells were stimulated with lipopolysaccharide (LPS) to increase expression of adhesion and costimulatory molecules on the surface of dendritic cells [35]. In the second intervention, T-cell ERK activity was inhibited as a way of attenuating the development of an immunological synapse between the T-cell and dendritic cell [36, 37]. The maximum optical force required to separate > 10 cell pairs for each condition, including the untreated without antigen cells, was measured after 120 second interaction time, the full data set is presented in Fig 6 and summarized in Table 1.

**Table 1:** The median relative interaction forces presented in Fig 5 measured after a 120 second interaction time between the T cells and dendritic cells.

	Unpulsed DC (-OVA)	Antigen pulsed DC (+OVA)
Unstimulated	5.3 ± 1.0 pN	11.4 ± 1.1 pN
LPS-stimulated	4.5 ± 0.6 pN	15.5 ± 0.7 pN
FR180204-treated	3.7 ± 0.5 pN	3.9 ± 0.5 pN

## Discussion

T-cells demonstrated a more than two-fold increase in interaction force with dendritic cells when the dendritic cells had been pulsed with a specific antigen and the cells allowed to interact for 30 seconds or 120 seconds compared to the unpulsed case when specific antigen was not present, see Figs 5 and 6. For example, after an interaction time of 120 seconds the relative interaction force between the cell pairs increased from 5.3 ± 1.0 pN to 11.4 ± 1.1 pN for the case when the specific antigen was present. These results confirm the sensitivity of the measurement technique to the presence of cognate antigen present on the dendritic cell and recognized by the T-cell.

Crucially, this antigen-dependent force is seen to increase further, from 11.4 ± 1.1 pN to 15.5 ± 0.7 pN, when dendritic cells are activated with LPS which is known to increase the surface expression of adhesion molecules and enhance T-cell activity. When the dendritic cells had been activated with LPS a 3.5 fold increase in cellular adhesion force was observed compared to the without antigen case, see Fig 6. Conversely, when T-cells were pre-treated with the ERK inhibitor, the interaction force of these T-cells with antigen-pulsed dendritic cells was significantly reduced, from 11.4 ± 1.1 pN to 3.9 ± 0.5 pN, to a level similar to cells interacting with un-pulsed dendritic cells (3.7 ± 0.5 pN), suggesting that preventing formation of the immunological synapse has an early impact on the development of interaction forces.

The minimum and maximum measurable forces, clear in the floor and ceiling levels in Figs 5 and 6, arise from the range of laser beam power and hence the optical trapping force available. The SLM, used to create the three-point trap, makes the optical system inefficient, as it is a diffractive optical element, and would be the obvious optic to replace in order to generate higher trapping powers in the future. If a single-point trap was required the SLM could be replaced with a galvanometer controlled mirror or if a triple-spot trap was required it would be possible to use 3 laser beams. The benefit of the SLM over these two other solutions is that it is reconfigurable and the position and number of traps can be altered in real-time. The data points on the maximum relative force of 17.6 pN relate to cell pairs where it was not possible to break the interaction force and separate the T-cell from the dendritic cell and for this reason we report the median of the data points as opposed to the mean. Calculating the mean would lead to a misleading representation of the data sets since the values of the outlying data points are not known. To calculate the median, only the values of the central data points are required making it more suitable for representing the data sets in study. This technique is best suited to measuring the initial interaction (i.e. the interaction that forms in the first few minutes of cell contact), as after this time the contact formed between the cells will be too great to be separated with the optical trap. For this reason, in this study we looked at the interaction after 30 or 120 seconds. Our results are in good agreement with atomic force microscopy and micropipette methods that report interaction forces in the range of pN to nN, although such approaches often require several minutes of contact time to generate detectable levels of force [38, 39].

## Conclusion

We have presented a non-invasive optical trapping approach for interrogating the early stage interaction forces between individual immune cell pairs. This approach directly traps the cells of interest removing the need to incorporate exogenous beads to the biological system. Using an optical approach avoids having any direct mechanical contact with the cells, as would be necessary with an AFM or micro-pipette approach, and removes the risk of disturbing the normal function of the cell. Here we use a triple-spot optical trap to trap the cell as a whole and prevent the cell re-orientating within the trap. In future this will be particularly relevant for applications of optical trapping that use the equipartition equation and Hookes’ Law to determine trap strength. These approaches rely on accurately tracking fluctuations in the trapped object’s position over time due to Brownian motion, any additional movement due to the object rolling around in the trap would produce incorrect results. When implementing a triple-spot trap it is worth noting that it can decrease the maximum trapping force available, for the experiments presented here the maximum trapping force decreased by approximately 20% (see Fig 4). Crucially we have demonstrated that this technique is sensitive to changes in interaction forces due to therapeutic intervention, demonstrating its future role in testing new immunotherapeutics that may attenuate cellular interactions, as well as use as a research tool to enhance our understanding of T-cell activation.
