# Correction: A minimally invasive optical trapping system to understand cellular interactions at onset of an immune response

**DOI:** 10.1371/journal.pone.0196266

**Published:** 2018-04-17

**Authors:** David G. Glass, Niall McAlinden, Owain R. Millington, Amanda J. Wright

The full name of the funder is missing from the Funding section. The correct funding information is as follows: This work was supported by the Engineering and Physical Sciences Research Council [grant number EP/H024891/1]. The funder had no role in study design, data collection and analysis, decision to publish, or preparation of the manuscript.
